# Enthesis Healing Is Dependent on Scaffold Interphase Morphology—Results from a Rodent Patellar Model

**DOI:** 10.3390/cells11111752

**Published:** 2022-05-26

**Authors:** Carlos J. Peniche Silva, Sebastian A. Müller, Nicholas Quirk, Patrina S. P. Poh, Carla Mayer, Antonella Motta, Claudio Migliaresi, Michael J. Coenen, Christopher H. Evans, Elizabeth R. Balmayor, Martijn van Griensven

**Affiliations:** 1 cBITE, MERLN Institute for Technology-Inspired Regenerative Medicine, Maastricht University, 6229 ER Maastricht, The Netherlands; m.vangriensven@maastrichtuniversity.nl; 2 Musculoskeletal Gene Therapy Laboratory, Rehabilitation Medicine Research Center, Mayo Clinic, Rochester, MN 55905, USA; sebastian23mueller@gmail.com (S.A.M.); nicholas.paul.quirk@gmail.com (N.Q.); coenen.michael@mayo.edu (M.J.C.); evans.christopher@mayo.edu (C.H.E.); erosadobalma@ukaachen.de (E.R.B.); 3 Department of Orthopedic Surgery, University of Basel, P.O. Box 4001 Basel, Switzerland; 4 Berlin Institute of Health at Charité–Universitätsmedizin Berlin, Julius Wolff Institute, 13353 Berlin, Germany; patrina.poh@bih-charite.de; 5 Department of General, Visceral and Minimally Invasive Surgery and Endoscopy, Dr. Lubos Kliniken Bogenhausen, 81679 Munich, Germany; carla.k.mayer@googlemail.com; 6 BIOtech Research Center and European Institute of Excellence on Tissue Engineering and Regenerative Medicine, Department of Industrial Engineering, University of Trento, 38123 Trento, Italy; antonella.motta@unitn.it (A.M.); claudio.migliaresi@unitn.it (C.M.); 7 Department of Orthopaedic, Trauma and Reconstructive Surgery, RWTH Aachen University Hospital, 52074 Aachen, Germany

**Keywords:** enthesis, scaffold, multiphasic, silk fibroin, tendon

## Abstract

The use of multiphasic scaffolds to treat injured tendon-to-bone entheses has shown promising results in vitro. Here, we used two versions of a biphasic silk fibroin scaffold to treat an enthesis defect created in a rat patellar model in vivo. One version presented a mixed transition between the bony and the tendon end of the construct (S-MT) while this transition was abrupt in the second version (S-AT). At 12 weeks after surgery, the S-MT scaffold promoted better healing of the injured enthesis, with minimal undesired ossification of the insertion area. The expression of tenogenic and chondrogenic markers was sustained for longer in the S-MT-treated group and the tangent modulus of the S-MT-treated samples was similar to the native tissue at 12 weeks while that of the S-AT-treated enthesis was lower. Our study highlights the important role of the transition zone of multiphasic scaffolds in the treatment of complex interphase tissues such as the tendon-to-bone enthesis.

## 1. Introduction

The enthesis is a fibrocartilaginous tissue that connects tendons and/or ligaments to bone. In particular, the tendon-to-bone enthesis is an interphase tissue that features a structural gradient of extracellular matrix (ECM). The opposing gradients of collagen molecule alignment and mineralization present at the enthesis allow the smooth stress transfer from tendon to bone [1,2]. This insertion site can be anatomically described as a succession of four different tissues/zones with region-specific cell types and mineral content [1]: tendon, non-mineralized fibrocartilage, mineralized fibrocartilage, and bone [2]. This structural complexity is essential for the enthesis’ function in the body; it allows the transfer of mechanical loads between tendon and bone tissue and reduces stress at the insertion site. Regrettably, once the tendon-to-bone enthesis is damaged, its native structure is often not regenerated, resulting in scar formation, with poor mechanical properties and high rupture recurrence rates [2,3,4,5]. This damage often needs surgical repair, in which the injured tendon is reattached to its binding site in the bone using sutures or bone anchors [3,6], which has proven to be insufficient for promoting the regeneration of the native enthesis structure [3,7,8,9], as it relies purely on the reincorporation of the tendon into the bone at the enthesis without promoting the regeneration of the native tendon-to-bone transition [3]. One additional complication associated with the surgical intervention is the subsequent mineralization of the tendon area, usually involving endochondral ossification [10,11], reported in animal studies [12,13] as well as patient treatments [14]. The latter described a significant percentage of patients suffering from tendon mineralization after open augmented repair of the Achilles tendon or reconstruction of the anterior cruciate ligament [14,15]. Tendon mineralization can result in pain and tendon weakness, increasing the probability of recurrent tendon/enthesis rupture in treated patients [10].

To overcome these limitations and to enhance the regeneration of the tendon-to-bone enthesis, tissue engineering strategies are increasingly growing in popularity [2,16,17,18,19,20]. The combination of cells, biomaterials, and growth factors has shown promising results in studies whereby bone, tendon, and the enthesis tissue have been engineered in vitro and in vivo [2,8,21,22,23]. In the case of the enthesis, special attention has been directed to the design of scaffolds that mimic its native structural properties and complexity [23,24,25,26].

One example describes the fabrication of a tri-phasic scaffold using different combinations of PLGA and bioactive glass. This material promoted zone-specific distribution of cells in vitro [24] and stimulated fibrocartilage-like tissue deposition at the interphase region when evaluated in vivo [19]. Additionally, other reports of multiphasic scaffolds for enthesis regeneration describe promising results only in vitro [27,28], while others evaluated their constructs in vivo by implanting the scaffold subcutaneously rather than treating an actual enthesis defect [19,29]. Thus, the in vivo evaluation of the functionality of such constructs for enthesis regeneration is still lacking.

Additionally, different manufacturing techniques and construct designs have been explored. For example, electrospinning is a widely used technique that allows the generation of fibers in the nanometer and micrometer range for the production of scaffolds [30,31,32]. However, it has been shown that the dense packing of fibers of electrospun scaffolds might result in poor cell infiltration and proliferation [33]. A similar limitation was reported by Lipner et al. when using an aligned electrospun PLGA scaffold with a mineral gradient in a rat model for supraspinatus tendon repair. As a result, the healing process was dominated by fibrosis and scar formation [34].

Although the fibrous morphology that is usually obtained in electrospun scaffolds mimics the fibrous morphology of tendon tissue, porous scaffolds mimic the 3D morphology of cartilage and bone tissue better [35,36,37].

Freeze-drying is a simple, cost-effective technique that allows the generation of porous scaffolds with a fair amount of control over the pore size and orientation [38,39]. We have previously demonstrated that by combining directional freezing, freeze-drying, and salt leaching, it is possible to obtain biphasic scaffolds with the desired morphological structure and mechanical properties to mimic both, the tendon and bony morphology characteristic of the native enthesis [23]. Furthermore, we demonstrated that the obtained silk fibroin multiphasic scaffold for enthesis repair showed excellent in vitro biocompatibility and functionality [23,36]. While designing our constructs, we exploited the formidable biomechanical properties and malleability of the silk fibroin, which allows the creation of scaffolds with high porosity without compromising the robustness of the construct. This is especially relevant for load-bearing scaffolds [23]. Additionally, this material shows a biocompatible, slow degradation rate due to proteolytic activity, that corresponds to the rate of new tissue deposition, which makes it ideal for tissue engineering applications [40,41]. Interestingly, we observed a major impact of the interphase morphology on the mechanical properties of the scaffold [23]. Additionally, the presence of an interconnected transition zone promoted tissue-specific gene expression such as *Col1a1*, *Col2a1*, *Col3a1* and *Sox9* along the scaffold in adipose-derived mesenchymal stem cells [23]. Our investigations and that of other groups pointed out a crucial role of the interphase morphology in enthesis scaffolds for cellular proliferation, cytoskeleton reorganization, and possibly, cell differentiation [18,42,43] in vitro. We hypothesize that the morphology of the transition zone in engineered scaffolds will also have a significant impact on the healing process of the enthesis in vivo. Furthermore, we expect that the biocompatibility, the good mechanical properties, and tailorable degradability of the silk fibroin will play a favorable role for the healing process of the enthesis. Therefore, in this study, we developed a new enthesis injury model in the rat patella and enthesis defects were treated with silk fibroin multiphasic scaffolds that featured two distinctive interphase morphologies, smooth and abrupt. The fabrication technique was built upon our previously reported methodology [23]. Treated animals were observed for up to 12 weeks, and tissue healing was assessed using gene expression, µCT, histology, and mechanical testing. We found that a smooth transition between the bone and tendon-like phases of the enthesis scaffold induced region-specific cell morphology and matrix deposition, leading to de novo enthesis-like tissue formation.

## 2. Materials and Methods

### 2.1. Biphasic Silk Fibroin Scaffolds

**Figure 1:**
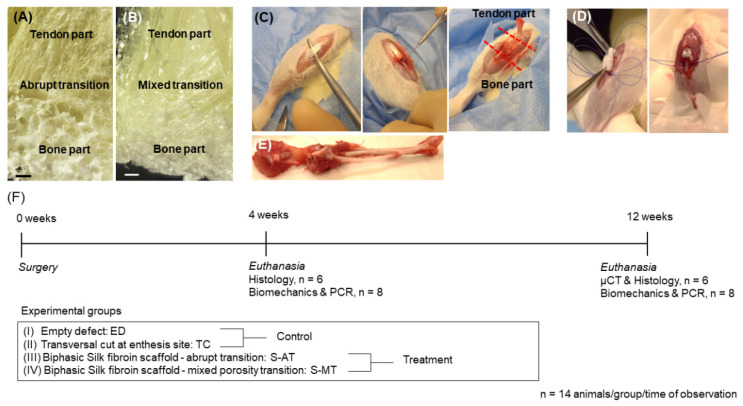
Schematic representation of the surgical approach. (**A**,**B**) Representative images of the biphasic silk fibroin scaffolds used for treatment, one with an interphase featuring an abrupt transition (S-AT) and one with an interphase presenting a mixed transition (S-MT; **B**). Scale bars represent 1 mm. (**C**) Exposure of the tendon and localization of the enthesis. Creation of the enthesis defect. (**D**) Scaffold implantation and fixation. (**E**) Representative image of the sample harvested at 12 weeks after surgery. (**F**) Timeline of surgery and subsequent times of observation. Specimens were collected for histology (n = 6) and biomechanics and PCR (n = 8). Samples at 12 weeks were also analyzed by µCT.

In the present study, a porous, biphasic silk fibroin scaffold featuring two distinct interface morphologies was used to treat a tendon-to-bone tissue defect at the enthesis site of a rat patella. The two different tendon-to-bone transitions recreated in the scaffolds featured either an abrupt phase transition or a mixed, smooth transition (Figure 1A,B). Furthermore, the scaffolds feature site-mimicking structures and properties at the bony and the tendon sites. The scaffolds were fabricated using a methodology previously published by our group [23]. A complete characterization of the biphasic silk fibroin scaffolds, which included biomechanics and in vitro performance has been published in [23,36].

Briefly, to produce the two versions of the biphasic scaffold, two different protocols were used. To obtain the scaffold with abrupt phase transition (hereafter termed as S-AT), the tendon zone of the scaffold, showing a lamellar-like structure of longitudinally oriented pores, was obtained first by directional freezing followed by freeze-drying. The pore sizes were controlled by adjusting the cooling rate of the 8% fibroin solution contained inside the molds. Thereafter, the tendon-like part was placed on top of a mixture of 8% silk fibroin solution containing 0.2 g of NaCl. After allowing for fibroin gelation to occur, the NaCl particles were removed from the scaffold by salt leaching with ddH_2_O. A porous structure resembling bone architecture remained. The entire construct was frozen and freeze-dried. The final product consisted of a biphasic scaffold with an abrupt transition between the two distinct phases (Figure 1A).

To obtain the scaffold with the mixed, smooth transition (hereafter termed as S-MT), the bony end of the scaffold showing randomly oriented pores was produced first by salt leaching and freeze-drying as described for the S-AT scaffold in the second stage. Then, the bony-like sponge was placed at the bottom of a mold and covered with an 8% fibroin solution. Directional freezing was induced followed by freeze-drying to produce a zone of vertically oriented pores (tendon-like zone). This two-step approach yielded a biphasic scaffold with a large interconnected area of mixed porosity between the two types of pore orientation (Figure 1B).

### 2.2. Rat Patellar Defect Model

Male Sprague Dawley rats (n = 112; Charles River Laboratories, Wilmington, MA, USA) weighing 400 g (100–115 d of age) were used in this study. The animals were allowed 48 h of acclimatization after arrival in the animal facilities. This study was approved by the Institutional Animal Care and Use Committee (IACUC, Protocol A00002479-17).

Prior to surgery, rats were placed in an anesthesia induction chamber with 3–4% isoflurane and an O_2_ flow of 1.5–2.0 L/min. After reaching a deep anesthesia state, rats were transferred to a heat pad (37 °C). Anesthesia was maintained using a nose cone with 2–3% isoflurane and an O_2_ flow of 1.5–2.0 L/min. Anesthesia depth was constantly monitored in addition to the respiration rate. Before starting the surgical procedure, rats received subcutaneous injections with Buprenex (0.6 mg/kg body weight) and cefazolin (50 mg/kg body weight). The right leg was shaved from the inguinal area up to the malleoli. The leg was disinfected with iodine and 70% ethanol. In supine position, the knee was moved to a 60° angle and the foot was fixed with tape to the operation table. A para-patellar longitudinal incision was made from the distal femur to the proximal tibia. The bursal tissue was lifted and opened using scissors, thereby exposing the patellar tendon. In order to keep the length of the patellar tendon similar to its original length after transection, a McLaughlin procedure was performed using a loop through a drill hole in the proximal tibia and around the patella. Two sutures were made on the medial and lateral side of the patellar tendon. Subsequently, the patellar tendon was detached from the tibial insertion using a scalpel. At the tibial insertion side, a defect in the bone was created using a Gigli saw. In the scaffold treatment groups (S-MT or S-AT), the scaffolds were oriented with the bony part to the tibial plateau and the tendon part towards the patellar tendon. The scaffolds were secured into the defect using sutures guided over the bony bridge using the mediolateral drill hole in the tibia. In addition to our scaffold-treated groups, two control groups were included. For the first control group (empty defect—ED), the defect was created in the enthesis as described above but nothing was inserted. The patellar tendon was sutured back over the bone defect. Thus, the sutures were directly going through the tendon and the bone. For the second control group (transversal cut—TC), a transversal cut at the tendon near the enthesis was performed without damaging the bone and no treatment was provided. The incision was closed in layers.

After surgery, the animals were placed in a 37 °C recovery chamber for 30 min before being placed back in their cages. Animals were allowed to freely move in their cages without any restriction or immobilization after surgery. The wounds were inspected daily for the first 5 d. After that, wounds were checked two times per week until the end of the observation period (12 weeks). The surgical approach and experimental design are summarized in Figure 1.

### 2.3. Mechanical Testing

For each of the four experimental groups, 14 animals were sacrificed at 4 and 12 weeks after surgery. Of these, 8 rats per sample group and time point of observation were used for the biomechanics and PCR studies. After sacrificing the rats, entire muscle–tendon–bone units were harvested (the distal fourth of the quadriceps muscle, the tendon, and the entire tibia including fibula).

Samples from the contralateral uninjured/healthy patellar enthesis were also harvested to be used as native tissue controls.

Before mechanical testing, the enthesis cross-sectional area (width × depth) and tendon length (muscle–tendon junction to bony insertion) were measured with an electronic CD-8 ASX Vernier caliper (Mitutoyo, Kawasaki, Japan). All measurements were conducted by a single operator. The sample length of interest was calculated as tendon length including the enthesis height.

Samples for mechanical testing were wrapped in a gauze soaked with saline and stored in a 15 mL Falcon tube at −20 °C until the day of testing [44]. Samples were thawed for 20–30 min at room temperature and kept moist with saline during the thawing period.

A custom-made, small biological specimen mechanical testing machine (Mayo Clinic, Rochester, NY, USA) with a MPL-2 50 lbf loadcell limit (Transducer Techniques, Temecula, CA, USA) and associated LabVIEW 2017 SP1 (National Instruments, Austin, TX, USA) was used for mechanical enthesis testing. The tibia was clamped by fixing the distal aspect rigidly whilst allowing neutral tendon alignment to the proximal attached muscle. The muscle was fixed by custom-built cryo-clamps [45]. Just before testing, muscles were frozen using dry ice powder to secure sample muscle attachments proximally. The temperature of proximal and middle tendon sections, as well as the enthesis, was monitored by a TrueRMS Supermeter multimeter (True RMS, Newport, RI, USA) during the freezing period to achieve optimal fixation. The optimal temperature for enthesis testing had been determined previously in a test study [44]. A mean temperature of −1 °C at the proximal tendon region assured sufficient muscle freezing in order to avoid muscle slippage. With a mean temperature of 10 °C at the enthesis region, mechanical testing could be performed reproducibly while the enthesis was not frozen. Samples were preloaded to 3 N prior to 200 mm/min failure tensile test [44]. Force–elongation curves were recorded, from which ultimate load (force at failure (N)), ultimate strain (elongation at failure/tendon length (%)), and tangent modulus ([force at failure/enthesis cross sectional area]/[ultimate strain] (MPa)) were calculated. Data were processed individually through Matlab 2016a (Mathworks, Natick, MA, USA), and compiled in Microsoft Excel 2010 (Microsoft, Redmond, WA, USA).

### 2.4. Micro-Computed Tomography (μ-CT) Analysis

Entheses samples (n = 6 per each of the 4 groups) harvested at 12 weeks after surgery were scanned using a Skyscan 1176 μCT (Bruker, Kontich, Belgium) at 90 kV and 277 µA. A 0.1 mm Cu filter was used. Images were acquired at a resolution of 35 µm. Image reconstruction was performed using NRecon v2.0.4 (Bruker, Kontich, Belgium), and analysis was performed using CTAn v1.13 (Bruker, Kontich, Belgium). Briefly, a region of interest (ROI) was selected by excluding the patella and tibia bones but including the area of the enthesis defect. Next, global thresholding of 90 to 255 was implemented for the binarization of images and further calculations using built-in algorithms in CTAn.

### 2.5. Histology

Collected specimens at 4 and 12 weeks after surgery (n = 6 per group and time point) were decalcified in 10% buffered EDTA (Sigma Aldrich, St. Louis, MO, USA), dehydrated in ethanol, and embedded in paraffin. Longitudinal cross-sections of the tendon-to-bone enthesis (thickness 7 µm) were sliced using the Leica RM 2165 microtome (Leica Biosystems, Nussloch, Germany). Subsequently, hematoxylin and eosin (H&E), and Safranin O stainings were performed. Briefly, after rehydration of the samples in descending ethanol series and distilled water, H&E staining was performed by incubating the slides with hematoxylin solution for 10 min and eosin for 2 min (Carl Roth GmbH, Karlsruhe, Germany). Thereafter, samples were dehydrated in ascending ethanol series, cleared with NeoClear-xylene substitute (Merck KGaA, Darmstadt, Germany), and mounted with UltraKit mounting media (Thermo Fisher Scientific, Landsmeer, The Netherlands). This staining allows the visualization of tissue microanatomy by staining the nuclear components of the cells purplish blue and staining structures such as elastic fibers, collagens and muscle fibers different shades of pink.

For Safranin O staining, the rehydrated samples were stained with hematoxylin solution for 10 min followed by 5 min staining with fast green solution (Sigma Aldrich, St. Louis, MO, USA), rinsing with 0.1% acetic acid, and further staining with 0.1% Safranin O solution for 10 min (Sigma Aldrich, St. Louis, MO, USA). Subsequently, the samples were dehydrated, cleared, and mounted as described before. Safranin O staining is specific for cartilage tissue. Proteoglycan-rich cartilage stains orange to red (also the endochondral ossifications), nuclei are stained black and the background stains green to blue.

Additionally, sections were stained with Masson–Goldner trichrome using a commercial kit (Carl Roth GmbH, Karlsruhe, Germany) according to the manufacturer’s protocol. When using this staining, dense collagen areas such as old bone appear dark green while cartilage and newly formed collagen-rich tissues appear pale green. Muscle and cell cytoplasm are stained red.

Images were taken with a Nikon DS-Ri2 camera mounted on a Nikon Ti Slide Scanner Microscope (Nikon Instruments Europe BV, Amsterdam, The Netherlands). In addition, sections were stained with picrosirius red to visualize collagens and their alignment by highlighting the natural birefringence of collagen fibers when exposed to polarized light. For the staining, sections were incubated for 1 h in a picric acid-saturated sirius red solution (Sigma Aldrich, St. Louis, MO, USA). Next, several washing steps were performed with acidified water. Finally, excess water was removed and sections were dehydrated further cleared in xylene, and mounted with a cover slip. Images were taken under polarized light microscopy in an Olympus IX83 inverted microscope (Olympus, Westborough, MA, USA) using the cellSens Dimension Desktop software v2.2 (Olympus, Westborough, MA, USA).

### 2.6. Gene Expression; qPCR Array

Immediately after processing the samples for biomechanics, the tissue was harvested for the qPCRs (n = 8 per group and time point) in RNALater^®^ solution (Sigma Aldrich, St. Louis, MO, USA). In order to guarantee that the quality of the RNA extracted from these samples would be as required by scientific standards, the equipment used for biomechanics, sample holder, and working table and surroundings were cleaned with ethanol 70% and RNAse AWAY^®^ (Sigma Aldrich, St. Louis, MO, USA) prior mechanical testing and between each sample. In addition to the samples of the treatment and control groups, samples from the healthy/native patellar enthesis of each rat were also included as controls (n = 8). The samples were homogenized with steel beads in TRIzol (Sigma Aldrich, St. Louis, MO, USA) using a TissueLyser II set to 3 min at 30 Hz (Qiagen GmbH, Hilden, Germany). Total RNA isolation was performed by phenol-chloroform extraction. The concentration and purity of the RNA were measured using a NanoDrop spectrophotometer (NanoDrop Tech. Inc., Greenville, SC, USA). A baseline for RNA purity values was set to be 1.7 for the ratio 260/230 and 1.8 for the ratio 260/280. Samples showing lower purity values than the baseline were further purified with the Monarch RNA cleanup kit (New England BioLabs GmbH, Frankfurt, Germany). The final purity values of all RNA samples used were, in every case, according to scientific quality standards. cDNA synthesis was performed using the RT^2^ First Strand Kit (Qiagen GmbH, Hilden, Germany) in a C1000 Touch Thermal Cycler (Eppendorf AG, Hamburg, Germany) following the manufacturer’s instructions. The Osteogenic RT^2^ Profiler PCR arrays (PARN-026Z) and the RT^2^ SYBR Green Mastermix were purchased from Qiagen (Qiagen GmbH, Hilden, Germany). PCR assays were performed in a CFX 96 Real-Time System thermocycler (Bio-Rad, Hercules, CA, USA) following the instructions of the manufacturer. The melting curves displayed a well-defined single distinct peak for each amplified gene, indicating that the amplified PCR products are single discrete species.

Ct values were exported to an Excel file following Qiagen’s instructions. This table was then uploaded to Qiagen’s data analysis web portal at http://www.qiagen.com/geneglobe, accessed on 6 June 2021. Samples were assigned to control (native tissue) and test groups (ED, TC, S-AT, and S-MT). Ct values were normalized against the arithmetic mean of the Ct values from the references genes *Ldha* and *Rplp1* included in the array set up.

The lower limit of detection was set to a Ct value of 35 using the “Set Cut-off” function in the web analysis portal. This function defines the upper limit of useful Ct values in calculating the fold change. All raw undetermined Ct values or Ct values greater than the set Ct cut-off value were automatically changed to the cut-off value. For the fold change calculations, genes that were not detected or showed a Ct value greater than the set cut-off value for both the control and test group samples were not considered. The data analysis web portal calculated fold change/regulation by means of the widely used delta delta Ct method (∆∆Ct), in which ∆Ct is calculated as the difference between the Ct value of a gene of interest and that of a reference gene followed by ∆∆Ct calculations (∆Ct(Test Group) − ∆Ct(Control Group)). The fold change was then calculated using the 2^(−∆∆Ct)^ formula. Treated data are displayed as fold regulation (FR) as this makes it easier to read and interpret. FR is the same as fold change (FC) for FC values > 1. For FC values < 1, FR is the inverse negative of FC. Additionally, a heat map was generated using Qiagen’s data analysis web portal that indicates with a gradient of color from green to black to red, the fold regulation of each gene compared to the expression in the native tissue. Black indicates same expression as native, grey indicates no expression, green indicates downregulation of the gene expression compared to the native tissue, and red indicates upregulation of the gene expression compared to the native tissue.

### 2.7. Statistical Analysis

The statistical analysis was performed using the GraphPad Prism 8.3.0 Software (GraphPad Software, San Diego, CA, USA). Measurable data are displayed as the mean ± SD.

For mechanical testing, the data on calculated mechanical properties are displayed through box plots (median, 1st, 3rd quartile ranges and outliers > 1.5 × IQR + Q 1/3). Statistical significance was determined by Kruskal–Wallis test and Dunn’s multiple comparisons test, with a *p* value < 0.05 indicating statistical significance. The μ-CT data were analyzed using one-way analysis of variance (df = 4; F = 4.139) followed by Dunnett’s multiple comparison test, with a *p* value < 0.05 indicating statistical significance.

## 3. Results

### 3.1. Rat Patellar Injury and Scaffold Treatment

All operations were successfully completed. All animals in all groups (i.e., scaffold abrupt transition, S-AT; mixed and smooth transition, S-MT; empty defect, ED; transversal cut, TC) remained healthy, assessed by twice-weekly checks, during the experimental period up to 12 weeks.

### 3.2. Mechanical Testing

**Figure 2:**
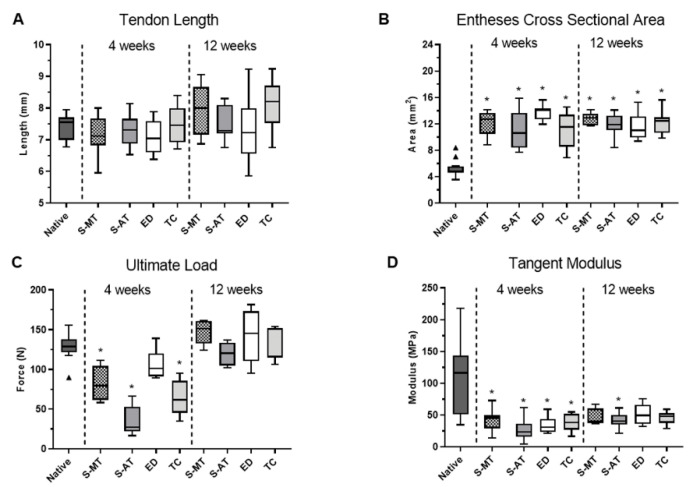
Measurements and mechanical testing of the samples at 4 and 12 weeks after surgery. (**A**) Tendon length, (**B**) enthesis cross sectional area, (**C**) ultimate load, and (**D**) tangent modulus. Tukey’s box plots show 1st and 3rd quartile, median and outliers from eight samples. Significant differences with respect to the native tissue are shown. Obtained p values are indicated as * for *p* < 0.05 and outliers are indicated by (▲).

The measurements of the associated tendon length at 4 and 12 weeks after surgery showed no significant differences between the scaffold-treated groups and the native tissue (Figure 2A), while the cross-sectional area measured in the scaffold-treated groups and the controls was significantly larger (*p* < 0.005) than that of the native enthesis (Figure 2B). Native tissue at 4 weeks after surgery showed the highest ultimate load compared to the S-MT, S-AT and TC groups (*p* < 0.005). The ED group was the exception, displaying values of ultimate load comparable to the native tissue as early as at 4 weeks after surgery. However, this result was not dependent on the tissue but solely on the suture material since the tendon was directly sutured back to the bone defect. In the scaffold-treated groups, the sutures did not go through the tendon nor through the scaffold. Thereby, the ED group seems to outperform the scaffold-treated groups. However, samples taken at 12 weeks after surgery from the scaffold-treated groups had similar ultimate loads as native tissue (Figure 2C). Similarly, the tangent modulus determined for the scaffold-treated groups at 4 weeks after surgery was lower (*p* < 0.05) than that of the native tissue (Figure 2D). Nevertheless, 12 weeks after surgery, the group treated with the S-MT scaffold showed a significant increase in the tangent modulus to values no longer statistically different to that of the native tissue, while the tangent modulus of the group treated with the S-AT scaffold remained lower (*p* < 0.01).

### 3.3. Micro-Computed Tomography (μ-CT) Analysis

**Figure 3:**
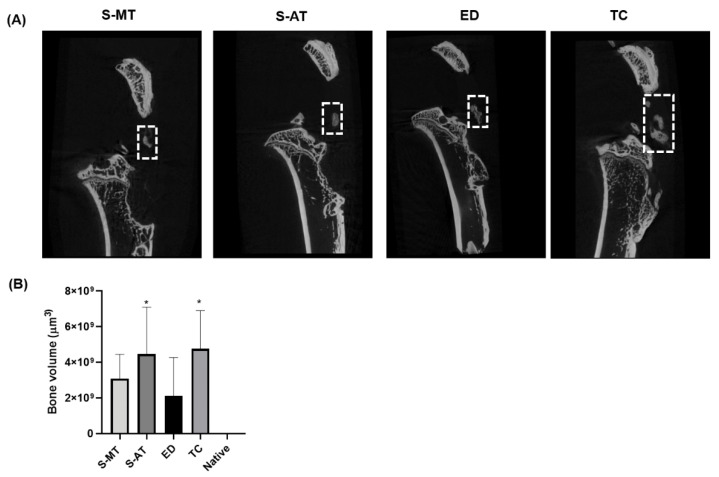
(**A**) µCT scans of the specimens harvested at 12 weeks after surgery. Detected ossification in the tendon tissue is indicated with dashed squares. (**B**) Comparison of bone volume (ossification) found in the tendon tissue for each of the treatment groups (S-AT with abrupt transition and S-MT with mixed transition) and the control groups (TC, transversal cut; ED, empty defect). Bone volume calculations are expressed relative to native tissue, with statistically significant differences denoted * *p* ≤ 0.05 from 6 samples.

µCT analysis performed at 12 weeks after surgery allowed the quantitative comparison of ectopic mineralization, an undesired complication during healing [10,46,47,48], occurring in the tendon area of the samples from the treatments and control groups relative to native tissue (Figure 3). This analysis revealed that the mineralization occurring in the tendon area of the samples treated with the S-MT scaffold was rather low and not of statistical significance. However, a significantly larger degree of mineralization was detected in the tendon of the samples from the TC and the S-AT groups (*p* < 0.05). Surprisingly, the control group ED showed a reasonably low degree of mineralization in the tendon.

### 3.4. Histology

**Figure 4:**
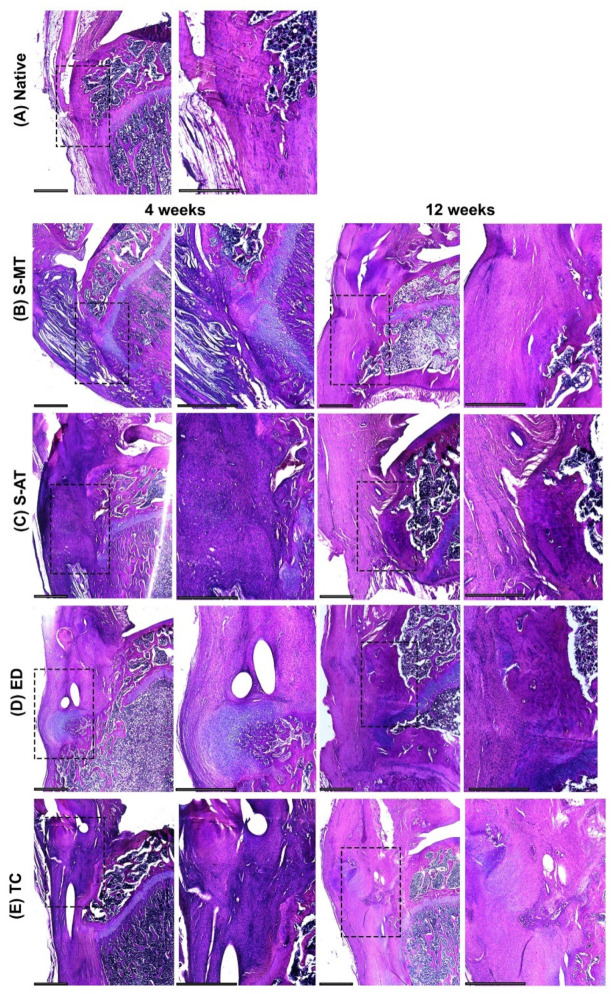
H&E staining. Representative images of the histological analysis of enthesis defects at the patellar region at 4 (left pairs) and 12 weeks (right pairs) after surgery, in (**A**) native enthesis, (**B**) S-MT with mixed transition, (**C**) S-AT with abrupt transition, (**D**) empty defect, and (**E**) transversal cut at enthesis site. Extracellular collagens and muscle fibers appear stained with different shades of pink. Dashed squares in the left pairs indicate the locations of the zoomed-in areas shown on the respective right. Scale bars = 800 µm.

**Figure 5:**
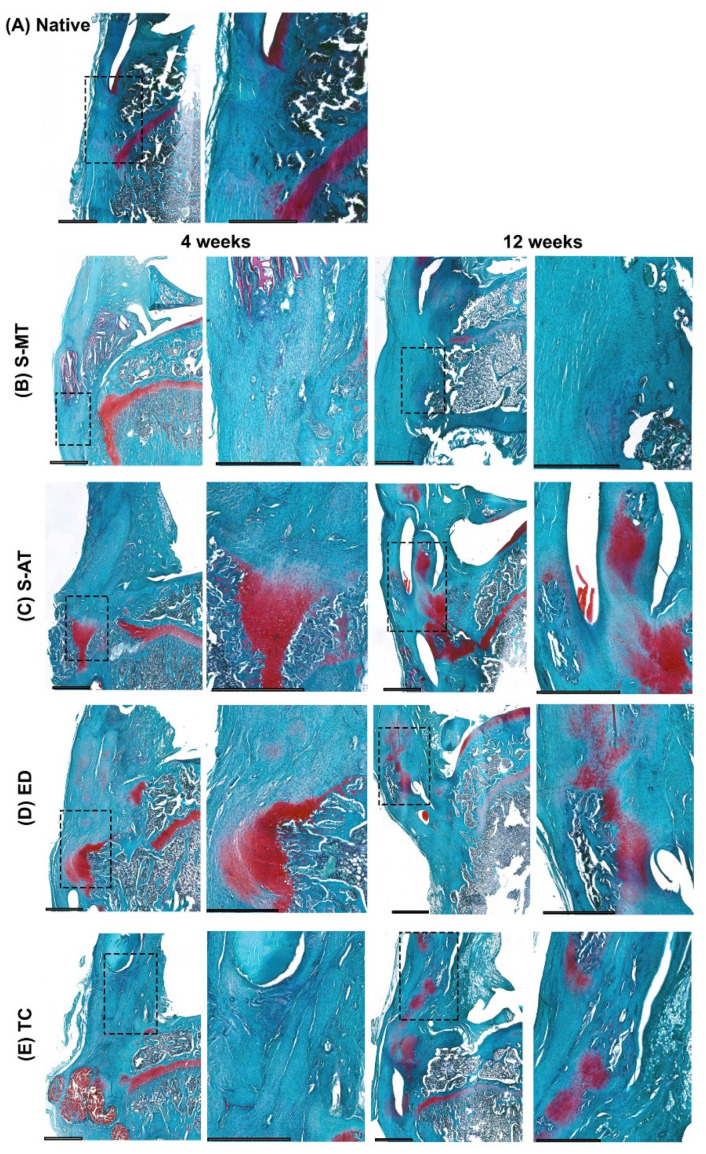
Safranin O staining. Representative images of the histological analysis of enthesis defects at the patellar region at 4 (left pairs) and 12 weeks (right pairs) after surgery, in (**A**) native enthesis, (**B**) S-MT with mixed transition, (**C**) S-AT with abrupt transition, (**D**) empty defect, and (**E**) transversal cut at enthesis site. Proteoglycan-rich cartilage appears red (here the orange to red color indicates endochondral ossification), and background appears blue. Dashed squares in the left pairs indicate the locations of the zoomed-in areas shown on the respective right. Scale bars = 800 µm.

**Figure 6:**
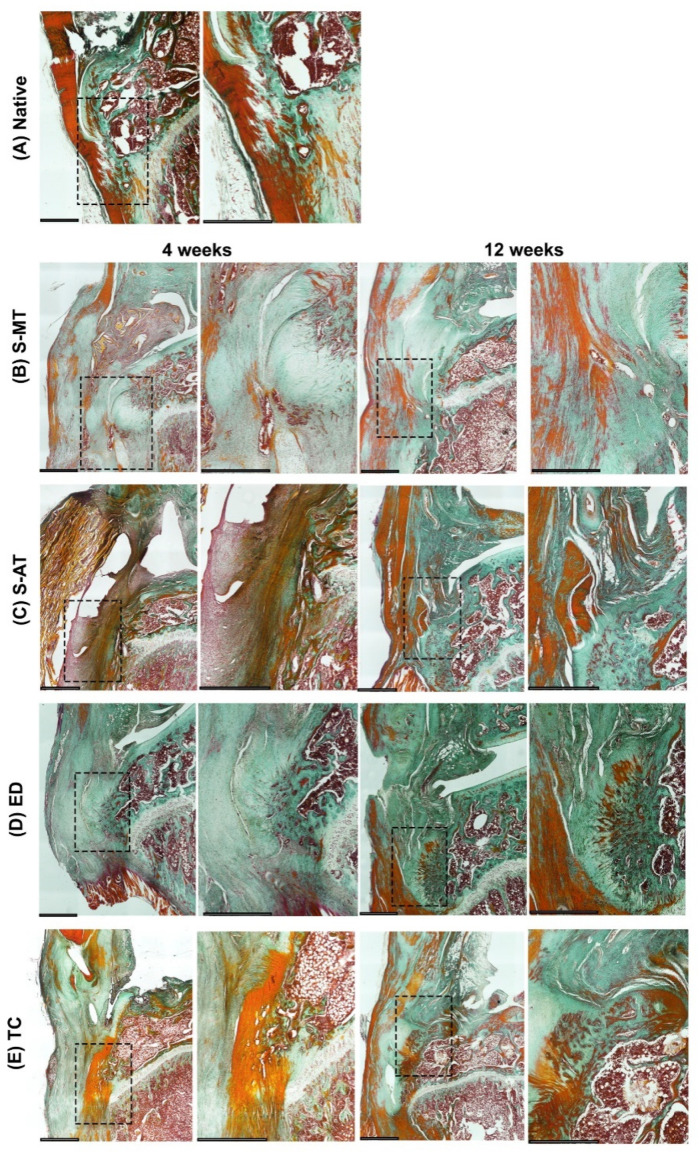
Masson–Goldner trichrome staining. Representative images of the histological analysis of enthesis defects at the patellar region at 4 (left pairs) and 12 weeks (right pairs) after surgery, in (**A**) native enthesis, (**B**) S-MT with mixed transition, (**C**) S-AT with abrupt transition, (**D**) empty defect, and (**E**) transversal cut at enthesis site. Dense collagen areas appear dark green, cartilage and newly formed collagen-rich tissue appears pale green. Muscle and cell cytoplasm are stained red. Dashed squares in the left pairs indicate the locations of the zoomed-in areas shown on the respective right. Scale bars = 800 µm.

The histology performed on the samples treated with the S-MT scaffold revealed a smooth transition from the tendon to the bone in the insertion site as early as 4 weeks after scaffold implantation (Figure 4B, Figure 5B and Figure 6B). Furthermore, the Masson–Goldner trichrome staining showed that at 12 weeks after surgery, the samples treated with the S-MT scaffold presented a fully restored native-like pattern of collagen deposition in the enthesis (Figure 6B). Moreover, H&E staining of the newly regenerated tissue in the S-MT samples revealed a longitudinal alignment of the Sharpey fibers in the tendon area of the enthesis similar to that observed in the native tissue (Figure 4B). Such a particular pattern was not observed in neither the samples of the S-AT nor in those from the control groups (Figure 3).

Safranin O staining revealed sites of cartilage deposition and endochondral ossification in the ED, TC, and S-AT groups that were absent in the S-MT group at both analyzed time points (Figure 5). The group treated with the S-AT scaffold developed some ossification in the shape of a bulky bony structure that protruded into the tendon area at the insertion site of the enthesis, as well as some isolated spots near the enthesis, where mineralization in the tendon region was visible (Figure 5C and Figure 6C). A similar ossification pattern at the insertion site was also visible in the control groups (Figure 5 and Figure 6). Additionally, both control groups developed scattered mineralization regions in the tendon area, particularly at 12 weeks after surgery.

**Figure 7:**
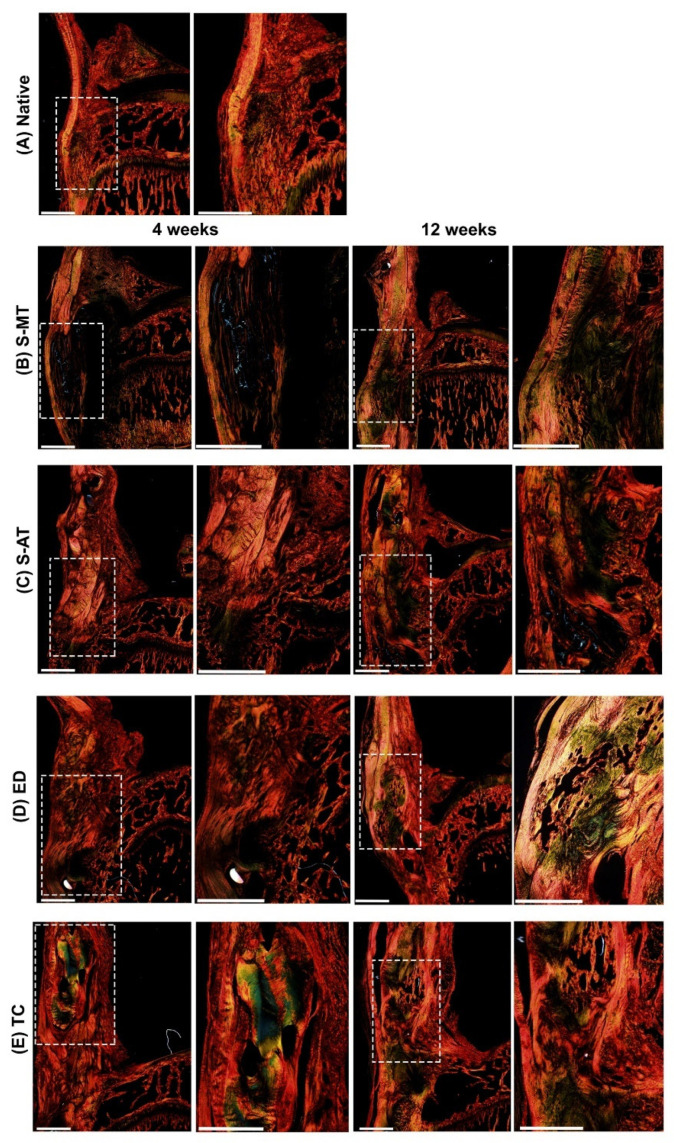
Picrosirius red staining. Representative images of the histological analysis of enthesis defects at the patellar region at 4 (left pairs) and 12 weeks (right pairs) after surgery, in (**A**) native enthesis, (**B**) S-MT with mixed transition, (**C**) S-AT with abrupt transition, (**D**) empty defect, and (**E**) transversal cut at enthesis site. Dashed squares in the left pairs indicate the locations of the zoomed-in areas shown on the respective right. Scale bars = 800 µm.

Picrosirius red staining of the collagen fibers in the samples treated with the S-MT scaffold (Figure 7B) confirmed the observations described above, highlighting a zone of longitudinally aligned collagen fibers in the tendon part of the enthesis at 4 weeks after surgery. At 12 weeks after surgery, these samples showed abundant staining of tightly packed collagen fibers where the scaffold had been implanted. This result indicated that the transition zone present in the S-MT scaffold effectively promoted the deposition and organization of the collagen fibers in the newly formed enthesis. In contrast, picrosirius red staining of the samples treated with the S-AT scaffold (Figure 7C) showed a lower presence of collagen fibers in the defect region compared to the S-MT-treated group at 4 weeks. The differences between the two treatment groups remained visible at 12 weeks after surgery, with the S-AT-treated group showing a mesh-like pattern of collagen deposition in the defect site rather than the aligned and more homogenous pattern observed in the S-MT-treated group. Furthermore, both control groups showed a more disorganized and heterogenous collagen deposition pattern in the defect zone (Figure 7D,E), more in line with tissue scarification than with the regeneration of the native enthesis tissue structure.

### 3.5. Gene Expression; qPCR Array

**Figure 8:**
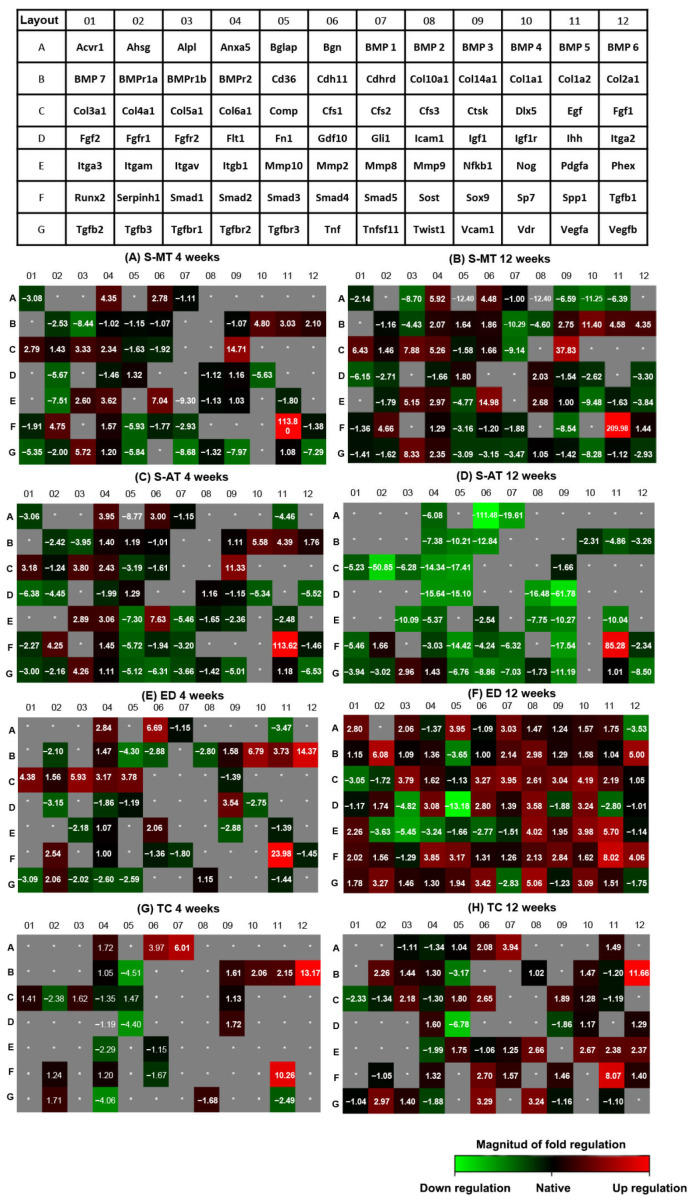
Gene expression in explanted samples at 4 weeks (left column) and 12 weeks (right column) after implantation (n = 8). (Top table) Plate layout with 84 genes investigated. Heat maps represent fold regulation of the gene expression compared to native tissue. Green indicates downregulation, red indicates upregulation, grey* indicates undetected expression and black indicates native levels of expression (= no regulation compared to native tissue). (**A**,**B**) S-MT, silk scaffold with mixed transition; (**C**,**D**) S-AT, silk scaffold with abrupt transition; (**E**,**F**) ED, empty defect; (**G**,**H**) TC, transversal cut.

The gene expression signature of 84 osteogenic and ECM-relevant genes was analyzed using RT-qPCR arrays. The fold regulation of the expression was determined with respect to the expression in the native tissue for genes with a Ct value ≤ 35. The cut-off for fold regulation was set at two fold. The top 10 upregulated and downregulated genes for each sample group are shown in Appendix A Table A1 and Table A2, respectively. The heat map shows the genes that were downregulated for each group compared to the native tissue in green. Conversely, the heat map shows upregulated genes compared to the native tissue in red. Additionally, black indicates the same expression as native tissue and grey indicates undetected genes/no expression. The samples treated with the S-AT and S-MT scaffolds showed similar patterns of gene expression at 4 weeks after surgery (Figure 8A,C). At this time point, key genes identified to play an important role in tissue remodeling, tendon and cartilage formation, and extracellular matrix deposition (e.g., *Col1a1*, *Col2a1*, *Col3a1*, *Col5a1*, *Col6a1*, *Ctsk*, *Mmp2*, *Serpinh1* and *Spp1*) were upregulated by several fold in both scaffold-treated groups compared to native expression. On the contrary, the osteogenic markers *Bmp2*, *Bmp6*, and *Bmp7* were not expressed in the samples treated with the S-MT and S-AT scaffolds while *Bmp-4* was downregulated by 4.5 fold in the S-AT group and not expressed in the S-MT group. In addition, the osteogenic marker *Runx2* was downregulated or expressed at levels similar to native in both treatment groups at this time point: by 2.3 fold in the S-AT-treated group and by 1.9 fold in the S-MT group.

Interestingly, at 12 weeks after surgery, the gene expression pattern of the samples treated with the S-MT scaffold was substantially different from that of the S-AT group (Figure 8B,D). At this time point, the samples treated with the S-MT scaffold showed a further increase in the upregulation of ECM-relevant genes (e.g., *Col1a1*, *Col2a1*, *Col3a1*, *Col5a1* and *Col6a1*) compared to the already high expression of those genes at week 4. Similarly, the expression of *Ctsk*, *Mmp2* and *Spp1* increased from week 4 to week 12 in magnitudes of 23.1, 7.9, and 110 fold, respectively. Additionally, at 12 weeks after surgery, the samples from the S-MT group showed the downregulation of many of the osteogenic genes included in the array (*Bglap*, *BMP2*, *BMP6* and *BMP7*).

In contrast, in the S-AT group, genes upregulated at 4 weeks (e.g., *Col1a1*, *Col2a1*, *Col3a1*, *Col5a1* and *Col6a1*) were strongly downregulated at 12 weeks after surgery. Moreover, the expression of *Ctsk*, *Mmp2* and *Spp1* was downregulated by 12.9, 10.2 and 28.3 fold, respectively, compared to at 4 weeks. Additionally, the expression of *Serpinh1* decreased in the S-AT-treated group to 1.7 fold but remained upregulated by 4.7 fold in the S-MT group at 12 weeks after surgery.

Both control groups exhibited a strong downregulation of the majority of the genes included in the array at 4 weeks after surgery. Of the 84 genes, 42 and 57 genes were not expressed in the ED and TC groups, respectively (Figure 8E,G). From genes detected within our set Ct cut-off value, of relevance was the upregulation of *Col2a1*, *Bgn*, and *Spp1* in the samples of both control groups, compared to native expression. Additionally, *Bmp1* was upregulated by 6.0 fold in the TC group while its expression remained low in the ED group at the same time point. Conversely, the expression of *Col1a1*, *Col3a1* and *Col5a1* was upregulated in the ED group at 4 weeks but downregulated in the TC group at the same time point. The overall gene expression of the TC group at 12 weeks after surgery was similar to that observed at 4 weeks, with a few of the non-expressed genes at 4 weeks showing a small upregulation at 12 weeks (Figure 8H). The expression measured in the ED group showed a pattern of expression close to that observed in the native tissue (black boxes in the heatmap), lacking values of (extreme) down- or upregulation that are typically observed in regenerating tissue. However, some osteogenic genes such as *BMP1*, *BMPr1a*, *Bglap* and *Runx2* were among the few upregulated genes in the ED group at 12 weeks after surgery.

## 4. Discussion

Interphase tissues, such as the enthesis, have a high incidence of injuries due to their anatomic role [8,17,49]. The reconstruction of such a complex tissue remains among the major current orthopedic challenges [3,8,48]. Among the many different approaches followed by tissue engineers to address enthesis regeneration, the utilization of biomimetic constructs to treat enthesopathies has shown promising results in many in vitro studies [20,23,26,50]. In a previous study, we investigated the impact of the morphology of the transition zone of a biphasic silk fibroin scaffold on cell proliferation and gene expression in vitro [23]. Following a similar approach but different techniques and materials, others have confirmed our previous observations that a biphasic construct presenting an aligned arrangement of fibers towards one end of the construct (tendon-like), and a disorganized pattern of fibers towards the opposite end (bone-like) can effectively promote cell alignment and organization, and gene expression in vitro, that mimics the tissue-specific gene expression and cell distribution and orientation observed in the healthy enthesis [51,52].

However, evidence of the potential application of these constructs in vivo is still scarce. For example, Spalazzi and collaborators investigated their developed multiphasic scaffold in vivo concerning the improvement of the healing of interphase tissues [19]. Although this in vivo study demonstrated the effectiveness of their construct’s design in stimulating matrix heterogeneity and fibrocartilage formation, it was conducted in an ectopic, subcutaneous athymic rat model, rather than in an orthotopic, injured enthesis model. The lack of in vivo studies exclusively addressing the use of biomimetic constructs to heal injured enthesis has been acknowledged as a challenge still to overcome [3,53]. We have tried to fill in part of that gap in the present study.

Here, we have demonstrated that the morphology of the transition zone of a biphasic scaffold plays a significant role in promoting and directing the enthesis regeneration in vivo. Our research demonstrates that the treatment of an enthesis defect with our S-MT scaffold improved enthesis regeneration, allowing the injured tissue to develop mechanical properties and histological features that resembled that of the native enthesis at 12 weeks after surgery. We have also made evident that the conventional suture method used to treat the ED group fails to regenerate a native-like enthesis tissue. The histological assessment of these samples clearly demonstrated that reattaching directly the tendon to the bone leads to endochondral ossification and the native, microscopic structure is not restored (Figure 3, Figure 4 and Figure 5).

Moreover, the analysis of the gene expression of the scaffold-treated groups revealed that, while both scaffolds were able to promote the expression of relevant entheses markers at 4 weeks after surgery, only the samples from the S-MT group sustained this upregulation at 12 weeks (Figure 8). Relevant genes for tendon and enthesis regeneration, such as *Col1a1*, *Col1a2*, *Col3a1*, *Col5a1*, *Col6a1*, *Bgn*, *Fn1*, *Serphinh1* and *Mmp2*, were upregulated several folds in the samples of the S-MT scaffold group compared to native and to the ED group (Figure 7). Additionally, the performed array included osteogenic genes such as *Bglap*, *Bmp2*, *Bmp6* and *Bmp7*, all of which were strongly downregulated in the S-MT group compared to the ED group, which is a wanted feature. This could well indicate lower osteogenic activity at the enthesis area in the S-MT group compared to the ED, which may favor the regeneration of the cartilaginous transition at the tendon-to-bone insertion site retaining low chances of endochondral ossification at the enthesis or tendon sites.

Collagens type 1, type 2, type 3, and type 10 are the most abundant collagens at the enthesis and they are responsible for the mechanical features of this tissue [54]. Our gene expression study revealed that the expression of many of these collagens was sustained for longer in the samples treated with the S-MT scaffold than in the samples from the S-AT group and the control groups. Additionally, *Col5a1*, *Col6a1* and *Bgn* were also upregulated at both time points of observation in the S-MT-treated groups. These ECM markers play a pivotal role during tendon healing by stabilizing the fibril structure of collagen type 1 in healing tendons [55,56]. Likewise, the S-MT group exhibited sustained upregulation of other ECM-relevant genes for at least 12 weeks after surgery. This includes the procollagen-specific chaperone *Serpinh1*, which plays a key role in the assembly of triple-helical procollagen molecules [57], as well as *Mmp2* and *Tgfbr1*, which are known to be essential for ECM remodeling and the tendon healing process of acute injury [58,59,60]. In contrast, a strong downregulation of ECM-relevant genes was observed in the S-AT group at 12 weeks. Together with the lower tangent modulus measured for the explanted samples of this group, these data might indicate that the abrupt transition between the two phases of the S-AT scaffold failed to stimulate the levels of gene expression required to achieve effective enthesis repair and native-like mechanical properties in vivo.

The very good performance of our S-MT scaffold was also demonstrated by the significantly lower presence of ossification at the insertion site of the tendon compared to the S-AT-treated group and the control groups (Figure 3). According to the µCT scans, the ED group also showed lower ectopic ossification than the S-AT in the tendon area. Nevertheless, ossification was still present. We should point out, however, that we observed high variability between bone volume formations within the same tested group. This variability is most likely associated with the set region of interest (ROI) that delimited the defect area. The ROI was set by excluding the patella and tibia and including only the area of the enthesis defect. To ensure that all samples were treated similarly, and to avoid biased ROI selection, the same ROI was applied to all the samples analyzed. This comes with the limitation where the boundaries of the ROI, in some instances, failed to exclude the totality of the patella and/or tibia due to minor differences in sample size, sample orientation, and/or shape of the defect. Although differences were minor, this managed to increase the variability in the calculation of bone volume. We acknowledge this as a limitation of the utilized method to measure bone volume, but we believe that a more accurate measurement in such samples was not practically feasible.

The histological assessment of the ED group showed that ossification in this group occurred ectopically, and such ossification was not visible in the histological assessment of the S-MT samples. Heterotopic bone formation and mineralization are detrimental phenomena during tendon and enthesis healing [10,46,47,48], for which no effective treatment has been yet developed [47]. In our study, ossification at the enthesis, as well as isolated mineralization spots in the tendon region, was observed in the histological assessment of the samples from the S-AT groups and the ED and TC control groups. In line with these observations, we identified an upregulation of *Bgn* and *Bmp1* in the samples of those groups at 4 weeks after surgery. In particular, the samples of the TC control group, which developed the largest amount of mineralization in the tendon region of the enthesis, showed sustained upregulation of *Bgn* and *Bmp1* at 4 and 12 weeks after surgery. In addition to the role in the tendon response to injury previously described, it has been observed that *Bgn* plays a crucial role in matrix mineralization and bone formation, possibly by regulating BMP signaling [61,62]. Moreover, BMP1, also known as the procollagen C-proteinase, is a proteolytic activator of TGFβ-1 that plays an important role in fibrosis and bone formation [63]. Previous studies have shown a link between the ossification of the tendon and the upregulation of osteogenic markers such as *BMP2*, *BMP4*, and *BMP7* [46]. Interestingly, we did not observe a significant upregulation of these genes in either our treatment or control groups at 12 weeks. However, we did observe a several-fold downregulation of the osteogenic genes *Bglap*, *BMP2*, *BMP6*, and *BMP7* in the S-MT-treated group compared to the ED control group.

The analyses presented here (biomechanics, histology, µCT scans, and gene expression signatures) of injured and treated entheses in vivo provide much-needed insight into the role of the transition zone of multiphasic scaffolds in the effective regeneration of the tendon-to-bone enthesis. Future studies might combine the optimized S-MT morphology of the transition zone in multiphasic scaffolds with different biomaterials and the use of coating agents (e.g., bioactive growth factors [36]) to further improve the regeneration potential of these constructs.

The strategy described in the present study to improve the healing of an injured tendon-to-bone patellar enthesis supports our initial hypothesis about the potential role of the interphase morphology of a construct to stimulate the regeneration of such an interphase tissue in vivo. The results described here show that silk fibroin may be a suitable material to produce multiphasic constructs to be used for enthesis regeneration applications in vivo. Additionally, we have demonstrated the potential of tailored tissue engineering strategies to treat entheses defects as a complement to the conventional surgical-repair approaches. Nevertheless, in order to improve our strategy even further, we recommend fine-tuning the process of the design and fabrication of biomimetic constructs to specifically meet the morphological and biomechanical properties of the many different interphase tissues in the body.

## Data Availability

The datasets generated and analyzed during the current study are available from the corresponding author on reasonable request. The PCR data generated in this study has been uploaded to the public data repository Gene Expression Omnibus (GEO) https://www.ncbi.nlm.nih.gov/geo/query/acc.cgi?acc=GSE188540, accessed on 11 November 2021.

## Appendix A

**Table A1:** Fold regulation of the expression of the top 10 upregulated genes for each group and time point of observation.

**4 Weeks**				**12 Weeks**			
**S-MT Scaffold-Treated Group**		**S-AT Scaffold-Treated Group**		**S-MT Scaffold-Treated Group**		**S-AT Scaffold-Treated Group**	
**Gene Symbol**	**Fold Regulation**	**Gene Symbol**	**Fold Regulation**	**Gene Symbol**	**Fold Regulation**	**Gene Symbol**	**Fold Regulation**
Ssp1	113.8	Spp1	113.6	Ssp1	209.98	Spp1	85.28
Ctsk	14.71	Ctsk	11.3	Ctsk	37.83	Tgfbr1	2.96
Mmp2	7.04	Mmp2	7.63	Mmp2	14.98	Serpinh1	1.66
Col1a1	4.8	Col1a1	5.58	Col1a1	11.4		
Serpinh1	4.75	Bgn	4.48	Col5a1	7.88		
Anxa 5	4.35	Col1a2	4.39	Col3a1	6.43		
Col5a1	3.33	Anxa 5	3.95	Col6a1	5.26		
Col1a2	3.03	Col5a1	3.8	Col1a2	4.58		
Col3a1	2.79	Col3a1	3.18	Bgn	4.48		
Bgn	2.78	Smad1	2.89	Col2a1	4.35		
**4 Weeks**				**12 Weeks**			
**ED Control Group**		**TC Control Group**		**ED Control Group**		**TC Control Group**	
**Gene Symbol**	**Fold Regulation**	**Gene Symbol**	**Fold Regulation**	**Gene Symbol**	**Fold Regulation**	**Gene Symbol**	**Fold Regulation**
Ssp1	23.98	Col2	13.17	Ssp1	8.02	Col2a1	11.66
Col2a1	14.37	Spp1	10.26	Col2a1	6.08	Spp1	8.07
Col1a1	6.79	Bmp1	6.01	Pdgfa	5.7	Bmp1	3.94
Bgn	6.69	Bgn	3.97	Twist1	5.06	Tnf	3.29
Col5a1	5.93	Col1a2	2.15	Col2a1	5.00	Twist1	3.24
Col3a1	4.38	Col1a1	2.06	Dlx5	4.19	Tgfb3	2.97
Comp	3.78	Anax5	1.72	Tgfb1	4.06	Smad4	2.7
Col1a2	3.73	Tgfb2	1.71	Nog	3.98	Nog	2.67
Igf1	3.54	Col5a1	1.62	Bglap	3.95	Mmp9	2.66
Col6a1	3.17	Ctsk	1.61	Cfs2	3.95	Cfs1	2.65

**Table A2:** Fold regulation of the expression of the top 10 downregulated genes for each group and time point of observation.

**4 Weeks**				**12 Weeks**			
**S-MT Scaffold Treated Group**		**S-AT Scaffold Treated Group**		**S-MT Scaffold Treated Group**		**S-AT Scaffold Treated Group**	
**Gene Symbol**	**Fold Regulation**	**Gene Symbol**	**Fold Regulation**	**Gene Symbol**	**Fold Regulation**	**Gene Symbol**	**Fold Regulation**
Tnfsf11	−8.68	Mmp10	−7.30	Bmp4	−11.25	Bgn	−111.4
Bmpr1	−8.44	Vgefb	−6.53	Cdhrd	−10.29	Col4a1	−50.85
Vcam1	−7.97	Fgf2	−6.38	Nog	−9.48	Igf1	−61.78
Itgam	−7.51	Tnf	−6.31	Cfs2	−9.14	Bmp1	−19.61
Vegfb	−7.29	Smad4	−5.72	Alpl	−8.70	Sox9	−17.54
Smad3	−5.93	Itga2	−5.52	Vdr	−8.28	Comp	−17.41
Tgfbr3	−5.84	Mmp8	−5.46	Bmp3	−6.59	Icam1	−16.48
Fgfr1	−5.67	Igf1r	−5.34	Bmp5	−6.39	Flt1	−15.64
Igf1r	−5.63	Tgfbr3	−5.12	Fgf2	−6.15	Smad3	−14.42
Tgfb2	−5.35	Vcam1	−5.01	Col10a1	−4.60	Col6a1	−14.34
**4 Weeks**				**12 Weeks**			
**ED Control Group**		**TC Control Group**		**ED Control Group**		**TC Control Group**	
**Gene Symbol**	**Fold Regulation**	**Gene Symbol**	**Fold Regulation**	**Gene Symbol**	**Fold Regulation**	**Gene Symbol**	**Fold Regulation**
Cd36	−4.30	Cd36	−4.51	Fn1	−13.18	Fn1	−6.78
Bmp5	−3.47	Fn1	−4.40	Itgav	−5.45	Cd36	−3.17
Fgfr1	−3.15	Tgfbr2	−4.06	Fgfr2	−4.82	Col3a1	−2.33
Tgfb2	−3.09	Vegfa	−2.49	Cd36	−3.65	Itgb1	−1.99
Nfkb1	−2.88	Col4a1	−2.38	Itgam	−3.63	Tgbr2	−1.88
Cdh11	−2.88	Itgb1	−2.29	Bmp6	−3.53	Igf1	−1.86
Col10a1	−2.80	Twist1	−1.68	Itgb1	−3.24	Col4a1	−1.34
Igf1r	−2.75	Smad4	−1.67	Col3a1	−3.05	Anxa5	−1.34
Tgfbr2	−2.60	Col6a1	−1.35	Tnfsf11	−2.83	Vcam1	−1.16
Tgfbr3	−2.59	Mmp2	−1.15	Ihh	−2.80	Vegfa	−1.10
